# Best Sensor Configuration and Accommodation Rule Based on Navigation Performance for INS with Seven Inertial Sensors

**DOI:** 10.3390/s91108456

**Published:** 2009-10-27

**Authors:** Cheol-Kwan Yang, Duk-Sun Shim

**Affiliations:** School of Electrical and Electronics Engineering, Chung-Ang University, 221 HukSuk-dong, Dongjak ku, Seoul, 156-756, Korea; E-Mail: ckyang92@empal.com

**Keywords:** fault detection and isolation, fault accommodation, inertial sensors, parity equation, probability of correct isolation

## Abstract

This paper considers the best sensor configuration and fault accommodation problem for inertial navigation systems which use seven inertial sensors such as gyroscopes and accelerometers. We prove that when six inertial sensors are used, the isolation of a double fault cannot be achieved for some combinations of fault magnitudes, whereas when seven inertial sensors are used, the isolation of any double fault can be achieved. There are many configurations which provide the minimum position errors. This paper proposes four configurations which show the best navigation performance and compares their FDI performances. Considering the FDI performance and the complexity of the accommodation rule, we choose one sensor configuration and provide accommodation rules for double faults. A Monte Carlo simulation is performed to show that the accommodation rules work well.

## 1. Introduction

The reliability of any system can be enhanced by fault detection, isolation, and accommodation (FDIA). FDI methods have been studied since the 1960s in various areas of engineering problems. The reviews [1-3] and books [4,5] and references therein introduce various methods of FDI and diverse applications in engineering problems.

To make them reliable and enhance their navigation accuracy, inertial navigation systems (INS) often use redundant sensors. Numerous studies have been performed on the use of redundant inertial sensors in FDI, and there are many FDI papers on hardware redundancy, such as those based on look-up tables and the squared error (SE) method [6,7], generalized likelihood test(GLT) method [8], optimal parity test(OPT) method[9], multiple parity vector method [10], and reduced-order parity method for double faults [11].

Yang *et al.* [10,12] suggested accommodation rules for single and double faults based on the error covariance of an estimated variable, which is related to the navigation accuracy of INS. These accommodation rules give decision criteria to determine whether faulty sensors should be excluded or not. Yang *et al.* [12] suggested accommodation rules for six inertial sensors. However, when six inertial sensors are used, a double fault can be detected, but the faults cannot be isolated in some cases. Accommodation rules for seven inertial sensors are suggested in [13], which is a conference version of this paper and the accommodation rules are obtained for the simplest sensor configuration among the four configurations which provide the best navigation performance.

The present paper proves that when six inertial sensors are used, the isolation of a double fault cannot be achieved for some combinations of fault magnitudes whereas, when seven inertial sensors are used, isolation can be achieved for any double fault. The configuration which shows the best navigation performance is not unique. Actually, there are many configurations which provide minimum position errors. This paper proposes the four best configurations from the navigational viewpoint and provides accommodation rules for double faults for one of them. For the four best sensor configurations, the probability of correct isolation (PCI) is obtained and compared to select the configuration for which the accommodation rules are obtained. Considering the FDI performance and the complexity of the accommodation rule, we choose one sensor configuration among the four suggested configurations and provide accommodation rules for double faults.

This paper is organized as follows. The sensor configuration and null space of the measurement matrix are explained in Section 2. The fact that seven inertial sensors should be used to isolate any double fault is proved, and the four best sensor configurations for navigation performance are given in Section 3. For these four sensor configurations, the PCIs are simulated and compared with each other in Section 3. The accommodation rules for a double fault for seven inertial sensors are given in Section 4. The simulation results and conclusions are given in Sections 5 and 6, respectively.

## 2. Sensor Configuration and Null Space of Measurement Matrix

### 2.1. Sensor Configuration

Consider a typical measurement equation for redundant inertial sensors such as their acceleration or angular rate:

(1) m(t)=Hx(t)+f(t)+ε(t)

where:

m(t) = [m_1_ m_2_ … m_n_]^T^ ∈ R^n^ : inertial sensor measurement.
H(t) = [h_1_ h_2_ … h_n_]^T^: n×3 measurement matrix of sensor configuration with rank(H) = 3.
x(t) ∈ R^3^ : triad-solution(acceleration or angular rate).
f(t) = [f_1_ f_2_ … f_n_]^T^ ∈ R^n^ : fault vector.
ε(t) ∼ N(0_n_, σI_n_) : a measurement noise vector with normal distribution(white noise), all sensors are assumed to have the same noise characteristics.
N(x, y): Gaussian probability density function with mean x and standard deviation y.

The triad solution x(t) in (1) can be obtained by the least square method from the measurement as follows:

(2) $$\hat{\text{x}}(\text{t})={{(\text{H}}^{\text{T}}\text{H})}^{-1}{\text{H}}^{\text{T}}\text{m}(\text{t})$$

**Figure 1.**
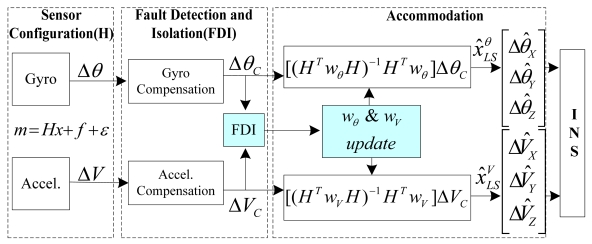
INS with redundant inertial sensor configuration and FDIA.

The navigation accuracy of INS depends on the estimation error of the triad solution x(t), as shown in Figure 1. The estimation error of the triad solution x(t) in (1) depends on the matrix H. Harrison *et al.* [14] mentioned the condition which provides the least estimation error of x(t) resulting in the best navigation performance.

#### Lemma 1 [14]

Consider the measurement Equation (1), where the matrix H ∈ R^n×3^ denotes the sensor configuration. When the eigenvalues of H^T^H are all equivalent to n/3, the sensor configuration provides the minimum estimation error of the triad solution x(t), which gives the best navigation performance.

### 2.2. Null Space of Measurement Matrix

A parity vector P(t) is obtained from the measurement using a matrix V as follows:

(3) p(t)=Vm(t)=Vf(t)+Vε(t)

where the matrix V satisfies:

(4) $$\text{VH}=0(\text{V}\in {\text{R}}^{(\text{n}-3)\times \text{n}}),{\text{VV}}^{\text{T}}=\text{I},\text{V}=[{\text{v}}_{1}\;{\text{v}}_{2}\cdots {\text{v}}_{\text{n}}].$$

The following Lemma shows the well-known singular value decomposition (SVD) result.

#### Lemma 2

Suppose that n>3. Every matrix H ∈ R^n×3^ with rank 3 can be transformed into the form H = UΛ = U[Σ 0]^T^= U_1_Σ

where U and Σ satisfy the following. UU* =U*U=I_n_, U=[U_1_ U_2_], U_1_ ∈ R^n×3^, U_2_ ∈ R^n×(n-3)^, Σ = diag{σ_1_, σ_2_, σ_3_} with σ_1_ > σ_2_ > σ_3_ > 0. ( )* denotes a complex conjugate transpose.

Measurement Equation (1) can be described as follows:

$$\text{m}={\text{U}}_{1}\sum \text{x}+\text{f}+\varepsilon$$

and the parity vector can be obtained by multiplying U_2_* on the left:

(5) $$p=U_{2}^{*}m=U_{2}^{*}(f+\varepsilon )$$

If we temporarily ignore the noise, we can obtain the null space projection of the fault f.

$${\hat{\text{f}}}_{\text{null}}={\text{U}}_{2}\text{p}={\text{U}}_{2}{\text{U}}_{2}^{*}\text{m}={\text{U}}_{2}{\text{U}}_{2}^{*}\text{f},$$

where U_2_U_2_* is the projector into the null space of the measurement matrix H. Thus, we can estimate the fault by using f̂_null_.

#### Remark 1

The matrix U_2_* in (5) can be used as V in (3). There are many solutions satisfying (4) for matrix V and the SVD method provides one of them.

## 3. Sensor Configuration for Seven Inertial Sensors

### 3.1. The Number of Sensors Required to Isolate a Double Fault

It is well-known that two faulty sensors can be isolated among six sensors. Gilmore *et al.* [6] mentioned that the symmetric configuration with six sensors arranged on dodecahedron enables self-contained failure isolation for up to two out of six sensors. This is correct when two faults occur at different times. However, in the case where two faults occur simultaneously, which is generally referred to as a double fault, the isolation of the two faults cannot be guaranteed, as will be proved in Theorem 1.

#### Theorem 1

Consider the measurement Equation (1) with n=6. Suppose that the magnitude of the faults differ from each other, i.e., f_i_ ≠ f_j_ for i ≠ j. Then, the isolation of the double fault cannot always be achieved for some combinations of f_i_ and f_j_.

##### Proof

Define a unit vector e_i_ for which only the i^th^ component is 1 and the other components are zero. Then we obtain double faults as follows:

(6) $${\hat{f}}_{jk}=U_{2}U_{2}^{*}(f_{j}e_{j}+f_{k}e_{k}),1\leq j<k\leq 6$$

The difference between f̂_jk_ and f̂_lm_

(7) $${\hat{f}}_{jk}-{\hat{f}}_{lm}=U_{2}U_{2}^{*}(f_{j}e_{j}+f_{k}e_{k}-f_{l}e_{l}-f_{m}e_{m})$$

can be zero for non-zero f_j_, f_k_, f_l_ and f_m_ since U_2_U_2_* has a maximum of three independent columns and, thus, there exist some combinations of f_j_, f_k_, f_l_ and f_m_ which make (7) zero. Therefore, a double fault cannot be isolated for some combinations of f_i_ and f_j_.

The simulation result for Theorem 1 can be seen in [15]. If we need to isolate a double fault for any combination of f_i_ and f_j_, seven sensors should be used.

#### Theorem 2

Consider the measurement Equation (1) with n=7. Then, the isolation of a double fault can be achieved for all combinations of f_i_ and f_j_.

##### Proof

Consider the double fault in (6), where U_2_ ∈ R^7×4^. The difference value:

$${\hat{\text{f}}}_{\text{jk}}-{\hat{\text{f}}}_{\text{lm}}={\text{U}}_{2}{\text{U}}_{2}^{*}({\text{f}}_{\text{j}}{\text{e}}_{\text{j}}+{\text{f}}_{\text{k}}{\text{e}}_{\text{k}}-{\text{f}}_{\text{l}}{\text{e}}_{l}-{\text{f}}_{\text{m}}{\text{e}}_{\text{m}})$$

cannot be zero for non-zero combinations of f_j_, f_k_, f_l_ and f_m_ since U_2_U_2_* has a maximum of four independent columns. Thus, a double fault can be isolated for any combination of f_i_ and f_j_.

Considering the result of Theorem 2, we need to use seven sensors to isolate double faults in any situation.

### 3.2. Four Sensor Configurations to Obtain Best Navigation Accuracy

**Figure 2.**
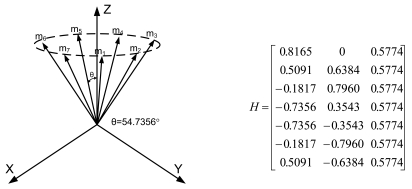
Configuration 1 of seven inertial sensors.

Lemma 1 gives the condition for the sensor configuration to provide the least estimation error of x in (2). Now we consider seven inertial sensors and there are many configurations which satisfy the condition, H^T^H = 7/3 I_3_. The configurations in Figures 2 through 5 all satisfy the condition, H^T^H = 7/3 I_3_.

### 3.3. FDI Performances of the Four Sensor Configurations

In this section, we suggest an appropriate sensor configuration in the case where seven inertial sensors are used, which takes into consideration simultaneously the navigation performance, FDI performance, and the complexity of the accommodation rule. This section considers the FDI performances for the four sensor configurations described in Section 3.2. Even though these four configurations all show the best navigation performance, their FDI performances are different from each other. For each configuration, we obtain the probability of correct isolation (PCI) with respective to each sensor. The PCI is obtained from 3,000 simulation runs and the PCI value in Table 1 denotes the average value of 30 raw PCIs. The GLT method[8] is used for FDI algorithm with false alarm rate of 1 %.

**Figure 5.**
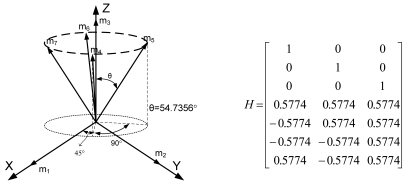
Configuration 4 of seven inertial sensors.

**Figure 6.**
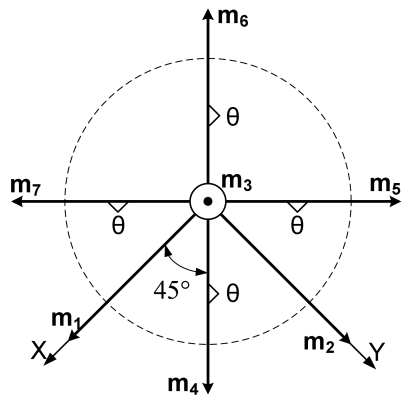
Configuration 4 redrawn.

The FDI performances of the four sensor configurations do not differ from each other very much. However, we can recognize that configuration 1 shows the worst FDI performance among the four configurations, while configurations 2 and 4 are better than configuration 3. Even though the magnitude of the fault varies, the PCI values of the four configurations show similar trends to those in Table 1. Actually configuration 4 shows only slightly better PCI performance than configuration 2. However, we chose configuration 2 after considering the complexity of the accommodation rule. Configuration 2 has four sets of accommodation rules, which are explained in detail in Section 4.2, while configuration 4 has 13. The number of different combinations of seven different sensors, taken two at a time, without repetition, is _7_C_2_ = 21. Figure 6 is redrawn for configuration 4 in Figure 5 and it shows symmetry; sensors 1 and 7 correspond to sensors 2 and 5, respectively, with respect to the plane consisting of sensors 3, 4, and 6. Among the 21 cases, eight are mirror images of the remaining 13 cases, as shown in Table 2. Because of this complexity, we chose configuration 2 instead of configuration 4.

## 4. Accommodation Rule for Seven Inertial Sensors

### 4.1. Accommodation Rule for Single and Double Faults

In this section, the results of [10,12] are used for the accommodation rules for single and double faults.

#### Category 0 [10]

When a single fault satisfies the following inequality $\left|f_{i}\right|\geq \frac{\sigma }{\left\|v_{i}\right\|}$, the faulty sensor should be excluded. The vector v_i_ is the i^th^ column of matrix V in (4).

#### Category I [12]

When double faults satisfy the following three inequalities:

-
${\text{f}}_{\text{i}}^{2}{\left\|{{(\text{H}}^{\text{T}}\text{H})}^{-1}{\text{h}}_{\text{i}}\right\|}_{2}^{2}+{\text{f}}_{\text{j}}^{2}{\left\|{{(\text{H}}^{\text{T}}\text{H})}^{-1}{\text{h}}_{\text{j}}\right\|}_{2}^{2}+2{\text{f}}_{\text{i}}{\text{f}}_{\text{j}}<{{(\text{H}}^{\text{T}}\text{H})}^{-1}{\text{h}}_{\text{i}},{{(\text{H}}^{\text{T}}\text{H})}^{-1}{\text{h}}_{\text{j}}>\;<\zeta_{1}$
-
${\text{f}}_{\text{i}}^{2}+{\text{f}}_{\text{j}}^{2}\frac{\left\{{\left\|{{(\text{H}}^{\text{T}}\text{H})}^{-1}{\text{h}}_{\text{j}}\right\|}_{2}^{2}-{\left\|{{(\text{H}}^{\text{T}}{\text{W}}_{\text{i}}\text{H})}^{-1}{\text{h}}_{\text{j}}\right\|}_{2}^{2}\right\}}{{\left\|{{(\text{H}}^{\text{T}}\text{H})}^{-1}{\text{h}}_{\text{i}}\right\|}_{2}^{2}}+\frac{2{\text{f}}_{\text{i}}{\text{f}}_{\text{j}}<{{(\text{H}}^{\text{T}}\text{H})}^{-1}{\text{h}}_{\text{i}},{{(\text{H}}^{\text{T}}\text{H})}^{-1}{\text{h}}_{\text{j}}>}{{\left\|{{(\text{H}}^{\text{T}}\text{H})}^{-1}{\text{h}}_{\text{i}}\right\|}_{2}^{2}}\langle \frac{\sigma^{2}}{{\left\|{\text{v}}_{\text{i}}\right\|}_{2}^{2}}$
- | f_j_ | < | f_i_ |

where $\zeta_{1}=\sigma^{2}\frac{{\left\|{\left({\text{H}}^{\text{T}}\text{H}\right)}^{-1}{\text{h}}_{\text{i}}\right\|}_{2}^{2}{\left\|{\text{v}}_{\text{j}}\right\|}_{2}^{2}+{\left\|{\left({\text{H}}^{\text{T}}\text{H}\right)}^{-1}{\text{h}}_{\text{j}}\right\|}_{2}^{2}{\left\|{\text{v}}_{\text{i}}\right\|}_{2}^{2}-\gamma }{{\left\|{\left({\text{H}}^{\text{T}}\text{H}\right)}^{-1}{\text{h}}_{\text{i}}\right\|}_{2}^{2}{\left\|{\left({\text{H}}^{\text{T}}\text{H}\right)}^{-1}{\text{h}}_{\text{j}}\right\|}_{2}^{2}{\text{D}}_{\text{ij}}}$ and γ = 2 < (H^T^H)^−1^h_i_, (H^T^H)^−1^h_i_ > < v_i_, v_j_ >, D_ij_ = ‖V_i_‖^2^‖V_j_‖^2^ − <V_i_, V_j_>^2^.

the two faulty sensors should not be excluded.

#### Category II [12]

When double faults satisfy the following three inequalities:

-
${\text{f}}_{\text{i}}^{2}{\left\|{\left({\text{H}}^{\text{T}}\text{H}\right)}^{-1}{\text{h}}_{\text{i}}\right\|}_{2}^{2}+{\text{f}}_{\text{i}}^{2}{\left\|{\left({\text{H}}^{\text{T}}\text{H}\right)}^{-1}{\text{h}}_{\text{j}}\right\|}_{2}^{2}+2{\text{f}}_{\text{i}}{\text{f}}_{\text{j}}<{\left({\text{H}}^{\text{T}}\text{H}\right)}^{-1}{\text{h}}_{\text{i}},{\left({\text{H}}^{\text{T}}\text{H}\right)}^{-1}{\text{h}}_{\text{j}}>\;<\zeta_{1}$
-
${\text{f}}_{\text{i}}^{2}+{\text{f}}_{\text{i}}^{2}\frac{\left\{{\left\|{\left({\text{H}}^{\text{T}}\text{H}\right)}^{-1}{\text{h}}_{\text{j}}\right\|}_{2}^{2}-{\left\|{\left({\text{H}}^{\text{T}}{\text{W}}_{\text{i}}\text{H}\right)}^{-1}{\text{h}}_{\text{j}}\right\|}_{2}^{2}\right\}}{{\left\|{\left({\text{H}}^{\text{T}}\text{H}\right)}^{-1}{\text{h}}_{\text{i}}\right\|}_{2}^{2}}+\frac{2{\text{f}}_{\text{i}}{\text{f}}_{\text{j}}<{\left({\text{H}}^{\text{T}}\text{H}\right)}^{-1}{\text{h}}_{\text{i}},{\left({\text{H}}^{\text{T}}\text{H}\right)}^{-1}{\text{h}}_{\text{j}}>}{{\left\|{\left({\text{H}}^{\text{T}}\text{H}\right)}^{-1}{\text{h}}_{\text{i}}\right\|}_{2}^{2}}\geq \frac{\sigma^{2}}{{\left\|{\text{v}}_{\text{i}}\right\|}_{2}^{2}}$
- | f_j_ | < | f_i_ |

the i^th^ sensor should be excluded, but not the j^th^ sensor.

#### Category III [12]

When double faults satisfy the following three inequalities:

-
${\text{f}}_{\text{i}}^{2}{\left\|{\left({\text{H}}^{\text{T}}\text{H}\right)}^{-1}{\text{h}}_{\text{i}}\right\|}_{2}^{2}+{\text{f}}_{\text{j}}^{2}{\left\|{\left({\text{H}}^{\text{T}}\text{H}\right)}^{-1}{\text{h}}_{\text{j}}\right\|}_{2}^{2}+2{\text{f}}_{\text{i}}{\text{f}}_{\text{j}}<{\left({\text{H}}^{\text{T}}\text{H}\right)}^{-1}{\text{h}}_{\text{i}},{\left({\text{H}}^{\text{T}}\text{H}\right)}^{-1}{\text{h}}_{\text{j}}>\;\geq \zeta_{1}$
-
$f_{\text{j}}^{2}<\zeta_{2}$
- | f_j_ | < | f_i_ |

where $\zeta_{2}=\frac{tr(A)}{tr(B)}$,

$$\begin{array}{c} \text{A}=\sigma^{2}{\left({\text{H}}^{\text{T}}\text{H}\right)}^{-1}\left\{\frac{1}{{\text{D}}_{\text{ij}}}[{\text{h}}_{\text{i}}{\text{h}}_{\text{j}}]\;\left[\begin{array}{cc} {\left\|{\text{v}}_{\text{j}}\right\|}_{2}^{2} & -{{\text{v}}_{\text{j}}}^{\text{T}}{\text{v}}_{\text{i}}\\ -{{\text{v}}_{\text{i}}}^{\text{T}}{\text{v}}_{\text{j}} & {\left\|{\text{v}}_{\text{i}}\right\|}_{2}^{2} \end{array}\right]\;\left[\begin{array}{c} {{\text{h}}_{\text{i}}}^{\text{T}}\\ {{\text{h}}_{\text{j}}}^{\text{T}} \end{array}\right]-\frac{1}{{{\left\|{\text{v}}_{\text{i}}\right\|}_{2}}^{2}}{\text{h}}_{\text{i}}{{\text{h}}_{\text{i}}}^{T}\right\}{\left({\text{H}}^{\text{T}}\text{H}\right)}^{-1}\\ \text{and}\\ \text{B}={\left({\text{H}}^{\text{T}}{\text{W}}_{\text{i}}\text{H}\right)}^{-1}{\text{h}}_{\text{j}}{{\text{h}}_{\text{j}}}^{\text{T}}{\left({\text{H}}^{\text{T}}{\text{W}}_{\text{i}}\text{H}\right)}^{-1} \end{array}$$

the i-th sensor should be excluded, but not the j-th sensor.

#### Category IV [12]

When double faults satisfy the following three inequalities:

-
${\text{f}}_{\text{i}}^{2}{\left\|{\left({\text{H}}^{\text{T}}\text{H}\right)}^{-1}{\text{h}}_{\text{i}}\right\|}_{2}^{2}+{\text{f}}_{\text{j}}^{2}{\left\|{\left({\text{H}}^{\text{T}}\text{H}\right)}^{-1}{\text{h}}_{\text{j}}\right\|}_{2}^{2}+2{\text{f}}_{\text{i}}{\text{f}}_{\text{j}}<{\left({\text{H}}^{\text{T}}\text{H}\right)}^{-1}{\text{h}}_{\text{i}},{\left({\text{H}}^{\text{T}}\text{H}\right)}^{-1}{\text{h}}_{\text{j}}>\;\geq \zeta_{1}$
-
${\text{f}}_{\text{j}}^{2}\geq \zeta_{2}$
- | f_j_ | < | f_i_ |

the two faulty sensors should be excluded.

#### Remark 2

For categories I through IV above, we consider only half of the first quadrant in two dimensional space. i.e., 0 ≤ θ ≤ π/4.

### 4.2. Accommodation Rule of Configuration 2

**Figure 7.**
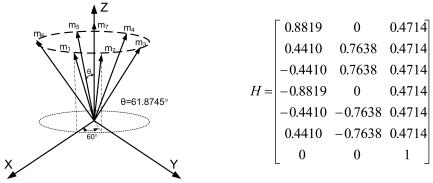
Configuration 2 with seven inertial sensors.

In Section 3.3, we choose sensor configuration 2, as shown in Figure 7, considering the navigation performance, FDI performance, and the complexity of the accommodation rule, altogether.

In this case, the measurement matrix H is given in (8) and has the following relations:

$${\text{H}}^{\text{T}}\text{H}=7/3{\text{I}}_{3},{\left\|{\text{h}}_{\text{i}}\right\|}_{2}=1,{\left\|{\text{v}}_{\text{i}}\right\|}_{2}=0.7559(i=1,2,\ldots ,7)$$

**Figure 8.**
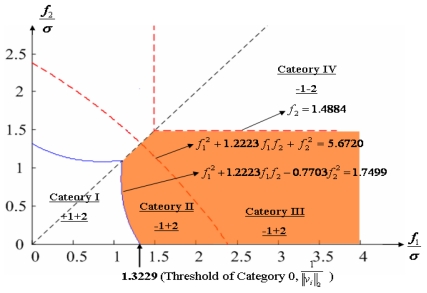
Region of categories I through IV for 1^st^ and 2^nd^ faulty sensors for configuration 2.

Configuration 2 in Figure 7 has six sensor input axes (i=1, …, 6) on the cone with angle 61.8745° from the Z-axis and one sensor input axis (i=7) on the Z-axis. There are four kinds of combinations of double fault. Three double fault combinations take place on the cone (f_i_, i=1, …, 6) : adjacent double faults (f_i_ and f_i+1_), double faults skipping a sensor (f_i_ and f_i+2_), and double faults skipping two sensors (f_i_ and f_i+3_). The other double fault combination takes place between the Z-axis (f_7_) and one sensor on the cone (f_i_, i=1, …, 6). For simplicity, we call these faults the 1^st^ and 2^nd^, 1^st^ and 3^rd^, 1^st^ and 4^th^, and 1^st^ and 7^th^ faults, respectively. The regions of Category 0 through Category IV in Section 4.1 are shown in Figures 8 through 11, respectively, where “-i” means that the i^th^ sensor should be excluded and “+j” means that the j^th^ sensor should be included.

### 4.3. Implementation of the Accommodation Rules: Update Matrix W

**Figure 12.**
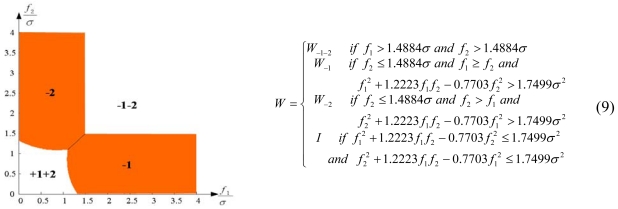
Accommodation rule for 1^st^ and 2^nd^ faulty sensors for configuration 2.

The accommodation rule is implemented by updating matrix W as in (9)–(12), where W_-i-j_ denotes the identity matrix with the i^th^ and j^th^ diagonal components having zero values, and W_-i_ denotes the identity matrix with the i^th^ diagonal component having zero value. Figures 12 through 15 show the accommodation rule in the first quadrant.

## 5. Simulations for Accommodation Rules

In this section, Monte Carlo simulations are performed 10,000 times for each double fault combination to confirm that the accommodation rules are correct. Seven identical sensors are used with configuration 2 as shown in Figure 7. For the measurement matrix H in (8), the matrix V satisfying VH = 0 and VV^T^ = I can be obtained by using the SVD method as follows:

$$V=\left[\begin{array}{ccccccc} 0.3223 & -0.0842 & -0.5298 & 0.7189 & -0.2640 & -0.1464 & -0.0168\\ 0.1671 & 0.3628 & -0.4380 & -0.1871 & 0.6654 & -0.3495 & -0.2207\\ -0.2497 & 0.5564 & -0.2722 & 0.0001 & -0.2381 & 0.5899 & -0.3864\\ -0.6142 & 0.3510 & -0.1575 & 0.1398 & -0.0472 & -0.2826 & 0.6108 \end{array}\right]$$

where ‖v_i_‖_2_ = 0.7559 (i = 1,2, …, 7).

To show the navigation performance, the error covariance of the triad solution x is used. The covariance matrices are defined as follows:

(13) $${\text{C}}_{+\text{i}+\text{j}}={\text{E}[(\hat{\text{x}}}_{+\text{i}+\text{j}}-\text{x}){\left({\hat{\text{x}}}_{+\text{i}+j}-\text{x}\right)}^{\text{T}}]$$

(14) $${\text{C}}_{-\text{i}-\text{j}}=\text{E}[{(\hat{\text{x}}}_{-\text{i}-\text{j}}-\text{x}){\left({\hat{\text{x}}}_{-\text{i}-j}-\text{x}\right)}^{\text{T}}]$$

(15) $${\text{C}}_{-\text{i}+\text{j}}=\text{E}[({\hat{\text{x}}}_{-\text{i}+\text{j}}-\text{x}){\left({\hat{\text{x}}}_{-\text{i}+j}-\text{x}\right)}^{\text{T}}]$$

Matrix C_+i+j_ denotes the error covariance of x̂ including the i^th^ and j^th^ sensor outputs and C_-i-j_ the error covariance of x̂ excluding the i^th^ and j^th^ sensors, and so on. It is known that the minimization of the trace of the error covariance matrix provides the best navigation performance. The traces of the error covariance matrices (13)–(15) will be calculated and compared with each other for the four accommodation rules, which results are shown in Figures 17, 19, 21, and 23.

### 5.1. Double Fault of 1^st^ and 2^nd^ Sensors

**Figure 16.**
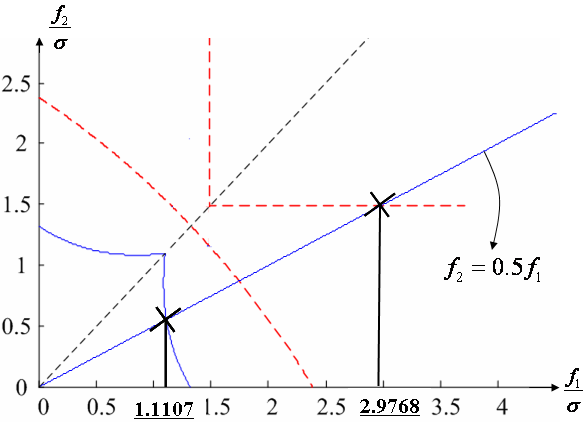
Accommodation rule for 1^st^ and 2^nd^ faulty sensors in Figure 12.

Suppose that the first and second sensors have a fault such that f(t) = [f_1_ f_2_ 0 0 0 0]^T^ with f_1_ and f_2_ being constants. Simulations are performed for each point on the linear line of f_2_ = 0.5 f_1_ as shown in Figure 16, and the measurement noise is white Gaussian with mean 0 and variance σ = 1.

**Figure 17.**
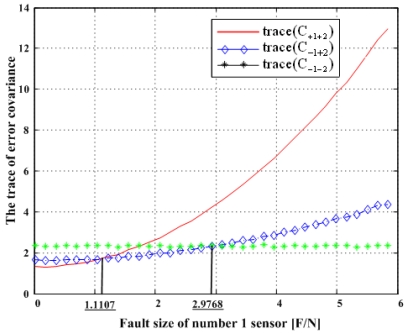
Trace(C_+1+2_), trace(C_-1+2_)and trace(C_-1-2_) with respect to fault magnitude in Figure 16.

Figure 17 shows the results of the accommodation rule for a double fault according to the fault size in Figure 16. When faults f_1_ and f_2_ belong to the region of Category I, the trace of C_+1+2_ is the minimum among the three traces. This means that when faults f_1_ and f_2_ belong to the region of Category I, the two faulty sensors should be used to obtain the minimum estimation error, in other words, the best navigation accuracy. When faults f_1_ and f_2_ belong to the region of Categories II or III, the trace of C_-1+2_ is the minimum, and when they belong to the region of Category IV, the trace of C_-1-2_ is the minimum. In Figure 16, the point at f_1_ = 1.1107 is the boundary between Category I and II, and the point at f_1_ = 2.2968 is the boundary between Category III and IV. These boundary points correspond to the crossover points between trace(C_+1+2_) and trace(C_-1+2_) and between trace(C_-1+2_) and trace(C_-1-2_), respectively, in Figure 17.

### 5.2. Double Fault of 1^st^ and 3^rd^ Sensor

**Figure 18.**
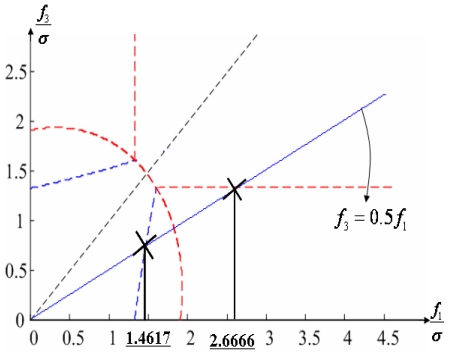
Accommodation rule for 1^st^ and 3^rd^ faulty sensors in Figure 13.

**Figure 19.**
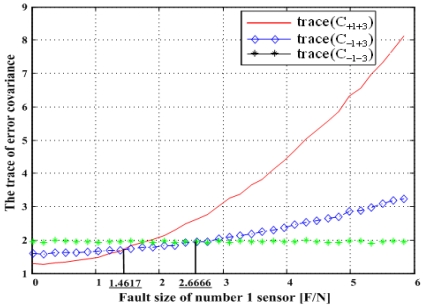
Trace(C_+1+3_), trace(C_-1+3_)and trace(C_-1-3_) with respect to fault magnitude in Figure 18.

Figure 19 shows the results of the accommodation rule for a double fault according to the fault size in Figure 18. The results are the same as those in Section 5.1.

### 5.3. Double Fault of 1^st^ and 4^th^ Sensors

**Figure 20.**
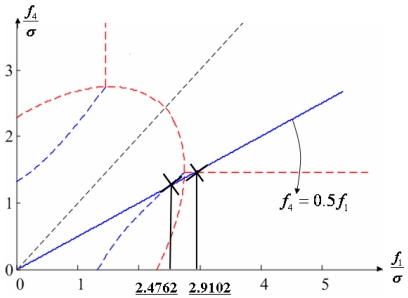
Accommodation rule for 1^st^ and 4^th^ faulty sensors in Figure 14.

**Figure 21.**
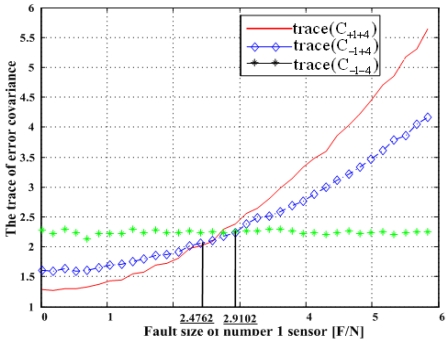
Trace(C_+1+4_), trace(C_-1+4_)and trace(C_-1-4_) with respect to fault magnitude in Figure 20.

Figure 21 shows the results of the accommodation rule for a double fault according to the fault size in Figure 20. The results are the same as those in section 5.1.

### 5.4. Double Fault of 1^st^ and 7^th^ Sensors

**Figure 22.**
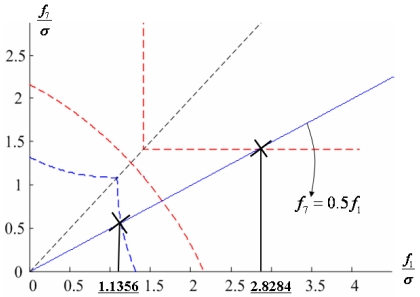
Accommodation rule for 1^st^ and 7^th^ faulty sensors in Figure 15.

**Figure 23.**
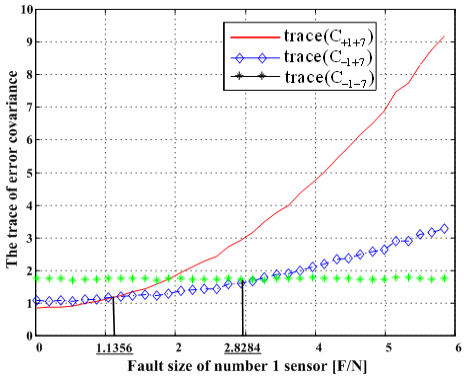
Trace(C_+1+7_), trace(C_-1+7_)and trace(C_-1-7_) with respect to fault magnitude in Figure 22.

Figure 23 shows the results of the accommodation rule for a double fault according to the fault size in Figure 22. The results are the same as those in Section 5.1.

## 6. Conclusions

For inertial navigation systems which use seven sensors, this paper proves that a double fault can be isolated for any combination of fault magnitudes. This paper suggests the four sensor configurations which provide the best navigation performance when seven sensors are used. The four sensor configurations are as follows: (1) cone configuration, (2) six sensor inputs on the cone surface and one sensor input on the center axis through the cone, (3) two sensors on the x and y axes, respectively, and the other five sensors on the cone surface with the z axis as center axis of the cone, (4) three sensors on the x, y, and z axes, respectively, and the other four sensors on the cone surface with the z axis as the center axis of the cone.

For these four configurations, the PCI is obtained for each sensor in order to compare their FDI performance. The Monte Carlo simulations indicate that configuration 4 shows the best PCI, but which is only slightly better than that of configuration (4). As explained in detail in Sections 3.3 and 4.2, configuration (2) has four sets of accommodation rules, while configuration (4) has 13. Thus, we chose configuration (2) after considering the complexity of the accommodation rule. For sensor configuration (2), four accommodation rules are obtained and a Monte Carlo simulation is performed. The Monte Carlo simulation shows that the suggested accommodation rules are correct and work well.

## Figures and Tables

**Figure 3.**
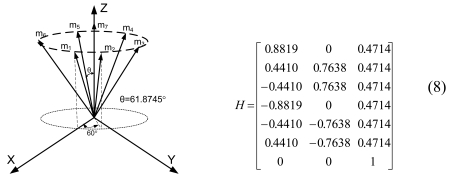
Configuration 2 of seven inertial sensors.

**Figure 4.**
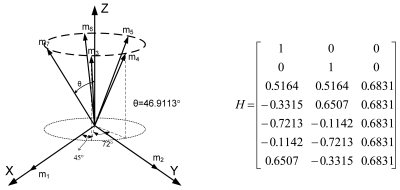
Configuration 3 of seven inertial sensors.

**Figure 9.**
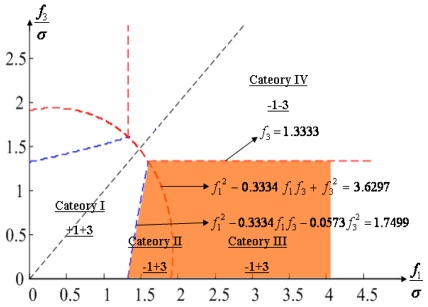
Region of categories I through IV for 1^st^ and 3^rd^ faulty sensors for configuration 2.

**Figure 10.**
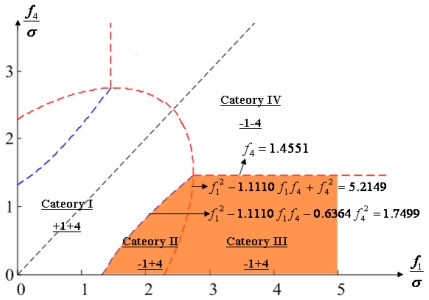
Region of categories I through IV for 1^st^ and 4^th^ faulty sensors for configuration 2.

**Figure 11.**
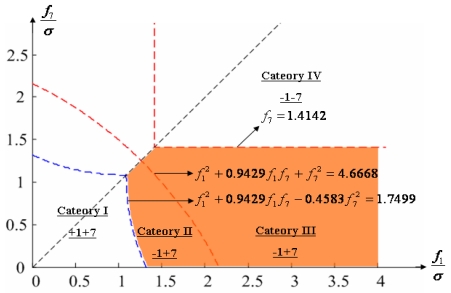
Region of categories I through IV for 1^st^ and 7^th^ faulty sensors for configuration 2.

**Figure 13.**
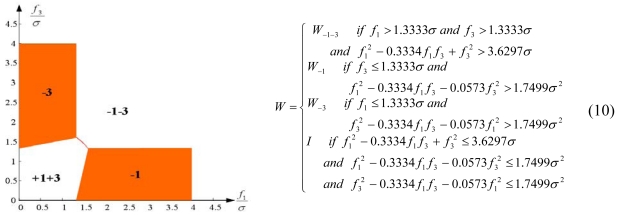
Accommodation rule for 1^st^ and 3^rd^ faulty sensors for configuration 2.

**Figure 14.**
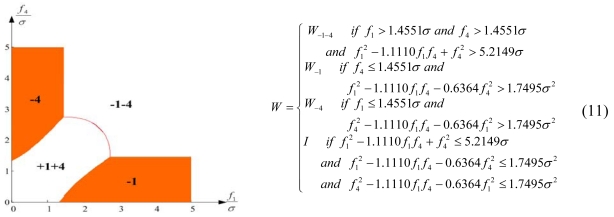
Accommodation rule for 1^st^ and 4^th^ faulty sensors for configuration 2.

**Figure 15.**
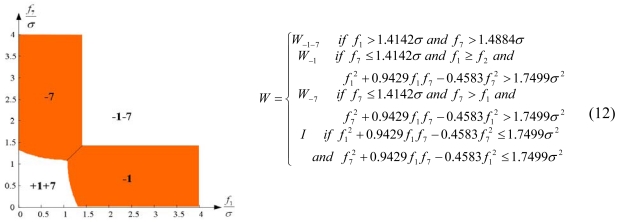
Accommodation rule for 1^st^ and 7^th^ faulty sensors for configuration 2.

**Table 1.** PCI value for single fault (f = 7σ, 3000 simulations, 30 averaged).

**Sensors**	**1st**	**2nd**	**3rd**	**4th**	**5th**	**6th**	**7th**
**Configurations**							
**Configuration 1**	0.964	0.963	0.965	0.965	0.965	0.964	0.963
**Configuration 2**	0.969	0.968	0.968	0.968	0.968	0.967	0.970
**Configuration 3**	0.966	0.967	0.969	0.966	0.966	0.966	0.967
***Configuration 4***	*0.967*	*0.968*	*0.969*	*0.969*	*0.969*	*0.968*	*0.969*

**Table 2.** Combinations of accommodation rules for Configuration 4 related to Figure 6.

	1	2	3	4	5	6	7	8	9	10	11	12	13
**Accommodation combinations**	**(1,2)**	**(1,3)**	**(1,4)**	**(1,5)**	**(1,6)**	**(1,7)**	**(3,4)**	**(3,5)**	**(3,6)**	**(4,5)**	**(4,6)**	**(5,6)**	**(5,7)**
**Mirror images**		**(2,3)**	**(2,4)**	**(2,7)**	**(2,6)**	**(2,5)**		**(3,7)**		**(4,7)**		**(6,7)**	
